# Computational Strategies Reshaping Modern Drug Discovery

**DOI:** 10.3390/molecules31020200

**Published:** 2026-01-06

**Authors:** Marco Tutone, Anna Maria Almerico

**Affiliations:** Dipartimento di Scienze e Tecnologie Biologiche Chimiche e Farmaceutiche (STEBICEF), Università degli Studi di Palermo, Via Archirafi 28, 90123 Palermo, Italy

Conventional drug development remains a protracted, costly, and high-risk endeavor, typically spanning 10–15 years from target identification to post-marketing surveillance. This multistage process encompasses target discovery and validation, hit identification and optimization, preclinical testing, clinical trials, regulatory approval, and ongoing safety monitoring. In this way, only 10–12% of candidates entering clinical trials reach regulatory approval. Despite being well established, this linear model of drug development is increasingly recognized as unsustainable. Each approved drug is estimated to cost over USD 2.5 billion, nearly 90% of candidates fail during clinical development, and meaningful breakthroughs, particularly for orphan diseases and targeted therapies, remain limited [1]. These shortcomings have catalyzed growing interest in computational approaches as transformative tools capable of accelerating discovery, reducing costs, and improving success rates in the search for viable drug candidates [2].

In traditional methods, once lead compounds are identified through high-throughput screening, structural optimization aims to enhance binding affinity, selectivity, bioavailability, and safety. This process is typically guided by structure–activity relationships (SAR) analysis [3]. Preclinical evaluation then assesses toxicity and efficacy in animal models [4], with only the most promising compounds advancing to human trials. Collectively, these stages underscore the inefficiencies of the conventional approach and highlight the urgent need for innovation to streamline drug development timelines [5]. Computational methods have emerged as a powerful catalyst in modern drug discovery, offering novel strategies to accelerate pharmaceutical innovation [6]. Traditional compound optimization methods are often labor-intensive and experimentally driven. In contrast, computational methods enable researchers to navigate vast chemical spaces efficiently, generating novel molecules with high affinity for specific biological targets in a fraction of the time [7].

AI-driven target identification and validation leverage large-scale biological datasets—including genomics, proteomics, and transcriptomics—to uncover disease-associated pathways, particularly in complex, polygenic disorders such as cancer and neurodegenerative diseases [8]. Deep learning models can predict biological activity and validate targets computationally, reducing reliance on costly and time-consuming laboratory assays [9]. One of the most transformative applications of computational methods lies in de novo compound generation. Deep learning architectures can design novel drug-like molecules that extend beyond existing chemical libraries while adhering to principles of chemical stability, drug-likeness, and bioactivity [10]. This capability significantly compresses early discovery timelines.

Computational methods also enhance drug optimization by predicting pharmacokinetic and pharmacodynamic properties, thereby improving candidate selection and reducing late-stage failures. By analyzing large datasets, Computational models can identify off-target effects and toxicity risks, enabling safer and more effective compound refinement [3]. Moreover, machine learning techniques such as random forests, support vector machines, and neural networks help prioritize the most promising candidates for downstream testing [11].

Beyond discovery, computational models are increasingly applied to preclinical and clinical development. Predictive models can assess toxicity and side effects without extensive animal or human exposure, while AI-assisted trial design improves patient recruitment, monitoring, and overall trial efficiency [12,13]. Generative AI is also a cornerstone of precision medicine, enabling therapies tailored to individual genetic and molecular profiles. By predicting patient-specific drug responses, AI supports personalized treatment strategies—particularly in oncology, where tumor heterogeneity demands highly individualized interventions [14,15,16]. Additionally, computer-driven virtual screening and drug repurposing allow rapid identification of new therapeutic uses for existing compounds. By mining large clinical and preclinical datasets, computer-assisted drug design can uncover previously unrecognized drug–target relationships, accelerating the availability of treatments for unmet medical needs [17,18,19].

In this context, this Special Issue consolidates a broad array of computational strategies—from structure-based methods like docking, molecular dynamics (MD) and free-energy profiling, to ligand-based techniques like pharmacophore modeling, similarity analysis, and QSAR (quantitative structure–activity relationships). This content curation sends a clear message: computational chemistry and bioinformatics are no longer niche—they are a central, mainstream pillar of modern drug discovery. As in earlier editions, the ambition is to blur the boundary between “in silico only” and “wet-lab + in silico” workflows, encouraging hybrid studies that combine computational predictions with experimental validation. The papers collected in this issue illustrate how computational tools are applied across a wide spectrum of real-world drug-discovery problems.
